# The Perspective of Using Ischemic Tolerance in Clinical Practice

**DOI:** 10.3390/biomedicines14010106

**Published:** 2026-01-05

**Authors:** Rastislav Burda, Marián Sedlák, Jozef Burda

**Affiliations:** 1 Department of Trauma Surgery, Faculty of Medicine, Pavol Jozef Šafárik University in Košice, Rastislavova 43, 040 01 Košice, Slovakia; 2 Slovak Academy of Sciences, 040 01 Košice, Slovakia

**Keywords:** ischemic tolerance, postconditioning, activated plasma, tourniquet, delayed neuronal death, apoptosis, CA1

## Abstract

Ischemic–reperfusion injury represents an extremely serious problem in the human population. It mainly affects the elderly population and currently used treatments have poor results. However, in nature there is a much more effective and relatively well-studied mechanism known as the ischemic tolerance phenomenon. If an organism is exposed to adverse conditions that do not destroy it, it responds by producing substances capable of protecting it from severe damage or death in the event of a repeated encounter with the same or a different dangerous environment. The problem with its use in the clinic is that its effectiveness decreases in the elderly and is practically lost with associated diseases and their concurrent treatment. Based on experimental animal studies and findings, it can be assumed that the activation of full tolerance—through successive exposure to two stressors in young, healthy individuals—will result in the formation of effectors of tolerance, which are spread throughout the body through the blood. Blood plasma thus activated and administered to a recipient who is unable to otherwise acquire tolerance, should be used as an immediate treatment for ischemia–reperfusion injury and a wide range of impending injuries in all individuals, since activated plasma contains effectors of ischemic tolerance. The purpose of this work is to show the possibilities of using ischemic tolerance in the clinical practice. Complete tolerance can be transferred from young, healthy, unmedicated donors to patients who have lost their ability to build tolerance in their own bodies.

## 1. Introduction

Living organisms in nature possess a rare and ultimately essential attribute: a relentless drive for survival. Even in the most sensitive population of cells or organisms, a certain percentage of resistant cells are usually found in the face of the same insult that proves fatal for most. This “miracle of nature” is known as ischemic tolerance. The first scientific evidence was presented by Murry and colleagues [1].

Establishing this protective phenotype in response to stress depends on a coordinated response at the genomic, molecular, cellular, and tissue levels [2].

If an organism is exposed to adverse conditions that do not destroy it, it reacts by producing substances capable of protecting it from severe damage or death in the event of repeated contact with a dangerous environment. The classic saying “what doesn’t kill you makes you stronger” is, in fact, true and holds under certain conditions. This ability is referred to as ischemic tolerance. Fascinatingly, this also works in reverse: if an organism or part of it finds itself in a potentially devastating situation, repeated similar stress may help it survive. Hence, the saying “a wedge drives out a wedge” is also applicable [3].

In addition, this mechanism is remarkable in that one type of stimulus can induce resistance to other types of stimuli—this is known as “cross-tolerance”. It may explain why individuals subjected to appropriate hypothermia (such as ice swimmers) exhibit resistance to various other forms of damage. Another valuable aspect of this mechanism is the so-called “remote tolerance”—the ability to induce protection across the entire organism, even when the initial stimulus affects only a part [4]. This is enabled by the protective substances of tolerance that circulate in the bloodstream [5].

In this review, we summarize the available experimental and clinical data of effectiveness of ischemic tolerance. It can be expected that ischemic tolerance can be used in the treatment of ischemic–reperfusion injury. The current partial knowledge of the mechanisms of ischemic–reperfusion injury does not provide the possibility of a comprehensive treatment of this multifactorial injury, while based on the available experimental works it should be expected that effectors of ischemic tolerance from “active” plasma could be used in the treatment of ischemic injury until the molecular identification of the effectors occurs.

## 2. Pathophysiology of Ischemia–Reperfusion Injury

Ischemia induces multifactorial changes in the organism, which are usually aggravated by changes caused by subsequent reperfusion. The cessation or strong reduction of oxygen supply, essential for aerobic oxidative phosphorylation in mitochondria, leads to a decrease in ATP. This causes a malfunction of Na^+^/K^+^-ATPase, and due to an increased influx of Na^+^ and water, the cell swells. The cell switches to anaerobic glycolysis, resulting in the accumulation of lactic acid and a decrease in pH [6].

Failure of both Na^+^/K^+^-ATPase and Ca^2+^-ATPase is manifested by potassium leakage from the cell and calcium accumulation in the cytoplasm, which leads to the activation of proteases and phospholipases. Mitochondrial damage and opening of the mitochondrial pore (MPTP) indicate cytochrome c leakage and the initiation of apoptosis [7]. If ischemia lasts too long, cell death occurs, mostly in the form of uncontrolled necrosis. Mild damage may lead to programmed cell death—apoptosis. In addition to necrosis as the resulting state of cell death, various states and processes leading to different forms and modes of cell death can be observed, and necrosis itself does not occur uniformly but in many ways (apoptosis, mitopsosis, necrosis, necroptosis, apoptosis, and delayed neuronal death) [8,9,10].

Ischemia leads initially to necrosis, but following reperfusion accelerates apoptosis, which occurs in almost 86% of cell death after prolonged ischemia [11,12,13,14].

Postischemic reperfusion causes complex cellular changes that may result in further damage (reperfusion lesion) or protective adaptations (in the case of conditioning). In the first minutes of reperfusion, so-called reactive hyperemia usually occurs. Tissue weakened by ischemia is unable to process the sudden oxygen influx, creating conditions for the formation of free radicals [15]. Explosive production of reactive oxygen species (ROS) is driven by the activity of NADPH oxidase, xanthine oxidase, and the mitochondrial electron transport chain [16,17]. The result is damage to lipids, proteins, and DNA [18].

Excessive accumulation of Ca^2+^ and ROS due to the opening of the mitochondrial permeability transition pore (mPTP) leads to decreased ATP synthesis and, in more severe cases, can result in apoptosis or necrosis [19,20]. During ischemia and reperfusion, protein synthesis is also significantly affected, although postconditioning can modulate this process in favor of cell survival. The cessation of protein synthesis during ischemia is a consequence of energy collapse. In the first minutes of reperfusion, ribosomes disintegrate from mRNA, stopping protein synthesis [21]. Disaggregation of polyribosomes is caused by phosphorylation of eIF2α, which blocks the formation of the translation initiation complex [22].

Reperfusion also allows protein synthesis to resume, but often with pathological consequences, such as the synthesis of pro-apoptotic proteins (e.g., Bax) [23]. At the same time, the accumulation of misfolded proteins in the endoplasmic reticulum activates the UPR (unfolded protein response) via the IRE1α, PERK, and ATF6 pathways [24,25].

In summary, it can be stated that during IR damage following pathological reaction occur: intracellular Ca and Na accumulation with rapid pH change, loss of mitochondrial membrane potential, occurrence of oxidative stress, massive ROS formation and uric acid generation, ROS-induced ROS generation, changes in NO metabolism, endothelial dysfunction, cytokines and chemokine signaling changes, expression of cell adhesion molecules, neutrophil tissues infiltration, platelet aggregation, and microembolization leading to autophagy and apoptosis [26].

## 3. Treatment Strategy of IR Injury

Many ways to reduce the extent of IR damage have been described so far. In recent years, attention has been paid to NO protective strategy (adenosine), [27] inhibition of apoptosis [28] and Ca^2+^ excess in the cell [29], antioxidants (SOD, catalase, N-ACC, vitamin E [30],) Na^+^H^+^ channel inhibitors [31], glutamate depletion [32,33], controlled reperfusion/reoxygenation [34], intermittent ischemia, neutrophil cell depletion [35], Aprotinin [36] poly (ADP-ribose) polymerase (PARP inhibitors) [37], blockade of the complement system, anesthetics, hypothermia, and hyperbaric oxygen therapy.

Currently, most attention is paid to the following strategies:

### 3.1. Pharmacological Agents

Glycogen synthase kinase-3β (GSK-3β) inhibitors is a multifunctional serine/threonine kinase which is involved in a variety of biological processes, including cell proliferation, apoptosis, and immune response. Inhibition or inactivation of GSK-3β provides protection against IR injury, making it a viable target for drug development. Though numerous GSK-3β inhibitors have been identified to date, the development of therapeutic treatments remains a considerable distance away. It is a potential target for future IRI therapy [38].

c-Jun N-terminal kinase (JNK) inhibitors has redox-centric mechanism, and its activation is involved in cell proliferation, migration, and invasion while inducing apoptosis and G1-phase cell cycle arrest. Its activation also has a future potential for treatment of brain tumor metastasis [39].

#### 3.1.1. Xanthine Oxidase Inhibitors

Allopurinol reduced oxidative stress which was the result of hypoxia/hyperoxia, as shown by decreased 8-isoprostane plasma concentration. However, during hypoxia, as well as hyperoxia, allopurinol administration resulted in a significant increase in autonomic control upon the heart as shown by increased standard deviation of all normal NN intervals with an increased vagal contribution [40]. Xanthine Oxidase Inhibitors have a promising effect in reducing oxidative stress in cardiac, lung, and brain cells [41], but their therapeutic potential in IRI remains under future investigation.

#### 3.1.2. Trimetazidine

Trimetazidine inhibits long-chain 3-ketoacyl-CoA thiolase; it changes energy reliance from fatty acid oxidation to glucose metabolism, and it reduces inflammation and the extent of IR damage [42].

#### 3.1.3. GSK-3β Inhibitors

GSK-3β inhibitors represent an adaptive response that might limit the extent of adverse remodeling in the aftermath of acute myocardial infarction, promote angiogenesis and reduce myocardial remodeling, especially after acute myocardial infarction, decrease apoptosis and fibrosis, and are very effective in tissue repair [43].

#### 3.1.4. JNK Inhibitors

JNK pathways are involved in diverse cellular processes, including growth regulation, transformation, and programmed cell death, and they are closely linked with cerebral IR injury [44], so inhibitors like SP600125 is promising for future therapy [45].

### 3.2. Anti-Inflammatory Therapies

#### 3.2.1. NF-κB Inhibitors

NF-κB is a transcription factor, which plays a role in inflammatory response during IR injury, and is involved in the release of pro-inflammatory factors and apoptosis of cardiomyocytes [46].

#### 3.2.2. RAGE (Receptor for Advanced Glycation End Products) Inhibitors

RAGE inhibition leads to lower expression of cytokines, which is responsible for endothelial dysfunction [47].

### 3.3. Regenerative Medicine

#### 3.3.1. Exosome-Based Therapy

Exosomes are membrane-bound extracellular vesicles (EVs) that are produced in the endosomal compartment of most eukaryotic cells. Exosomes derived from adipose-derived MSCs protect ischemic myocardium from I/R injury through the activation of Wnt/β-catenin signaling pathway [48]. Compared to MSCs, MSC-derived exosomes reveal many advantages such as non-immunogenicity, easy access, easy preservation, and extreme stability under various conditions [49]. Its usage is promising for future application [50].

#### 3.3.2. Micro RNA

MicroRNA, or rather some clusters, such as miR-17~92, can be highly expressed in endothelial cells during ischemia. Using targeted mRNA antagonists, better angiogenesis and functional repair of damaged tissue can be achieved in experiments [51].

None of the above-mentioned therapies have yet found significant use in practice.

## 4. Discussion—Ischemic Tolerance as a Therapeutic Strategy

Ischemic tolerance is a phenomenon where a sublethal ischemic insult protects against a subsequent, more severe ischemic event. This protective effect is an intrinsic mechanism where cells “learn” to tolerate stressful conditions.

Conditioning is a strategy whose goal is activation of ischemic tolerance. It should be applied as a brief interruption of blood flow, applied before (preconditioning), or during the early phase of reperfusion following an ischemic event (postconditioning). The onset of ischemia is unexpected; therefore, the use of preconditioning is very limited in clinical settings.

If ischemia–reperfusion changes are multifactorial, it logically follows that the treatment must also offer multifactorial protection. This is where the potential use of postconditioning arises. Compared to unprotected reperfusion, which itself causes further damage, postconditioning induces significant beneficial changes at the cellular and molecular levels.

Postconditioning significantly attenuates ischemia-induced neuronal death, suppresses the release of MnSOD and cytochrome c, and prevents caspase-3 activation [7,52]. It stabilizes the mitochondrial membrane through Akt/ERK activation and GSK-3β inhibition, preventing the opening of the mPTP [53]. It simultaneously increases the expression of Bcl-2 and HSP70, thereby blocking apoptotic pathways [54]. Postconditioning also enhances NO production via eNOS and improves vasodilation [55]. Experimental studies show that it also reduces the extent of damage in cerebral ischemia [56,57,58]. Moreover, a 5 min interruption of blood flow to the brain in a laboratory rat causes the death of up to 40% of the most sensitive CA1 hippocampal neurons, and 10 min without oxygen leads to the destruction of up to 70% of these cells. However, if a 10 min ischemia is applied within the right time window after a 5 min ischemia, there is no accumulation of damage; on the contrary, fewer than 10% of neurons die. This means that the combination of two otherwise lethal stimuli is required to save the cells [56,59].

Several important points must be noted here: after 5 or even 10 min of ischemia, not all brain cells die—and certainly not immediately. Even the most sensitive nerve cells in the brain, namely the neurons of the first layer of Ammon’s horn in the hippocampus, begin to die only after approximately 48 h, through a process known as delayed neuronal death [8,9]. This suggests that there is a substantial therapeutic window during which these cells can still be rescued. If each of these stresses can independently destroy 40% and 70% of neurons, respectively, their combination—when timed optimally—activates tolerance mechanisms that save the cells. Moreover, it does not matter whether the stronger or weaker stimulus is applied first.

Preconditioning, but especially postconditioning, can prevent the death of the most sensitive neurons in the brain (CA1 hippocampus), even at durations of oxygen deprivation exceeding the 5 min threshold, which is otherwise lethal without conditioning. Until recently, the 5 min limit was considered the point at which irreversible changes begin to develop. Our results show that CA1 hippocampal neurons can be rescued even after delayed postconditioning applied 48 h after a lethal 10 min brain ischemia [56,60,61]. This represents a therapeutic window of several hours to days in cases of reversible ischemia or hypoxia of the brain or the whole body.

**Figure 1:**
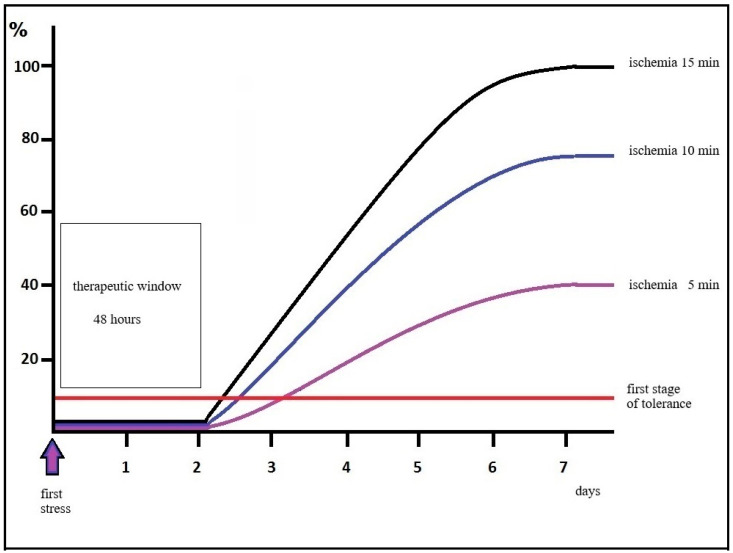
Diagram of the progression of CA1 neuron damage after transient brain ischemia in rats. Cells in the process of delayed neuron death begin to die only after two days which opens an exceptionally large therapeutic window. Even the first stress can cause dose-dependent damag.

**Figure 2:**
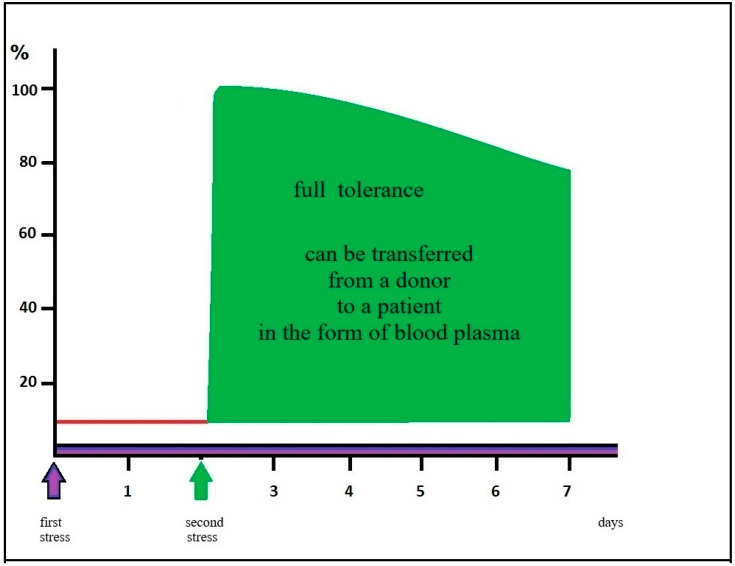
Tolerance induced by a second stress is able to prevent cell death occurring in the process of apoptosis. This condition also protects against ischemia-induced damage to muscle and brain after trimethyltin or kainic acid poisoning [56,62]. In young patients, we suggest the use of postconditioning. In people who have lost the ability to develop tolerance, activated blood plasma or its derivatives from donors should be used.

The delayed postconditioning effect described in the CA1 hippocampus is graphically illustrated in the following Figure 1 and Figure 2.

The effect of “remote conditioning” has come to the fore, where the stressor needs to be applied to only part of the body—most often a limb—to induce the effect. Stopping blood flow using an inflatable cuff for a single period of several minutes or through several repeated short (30 s to 5 min) periods of ischemia interrupted by equally long reperfusion times induces sufficient tolerance. Brief episodes of limb ischemia are safe and applicable to a wide range of potential organ damage, including both ischemic and toxic injury [62,63,64].

During the last three decades, a wide variety of ischemic conditioning strategies and pharmacological treatments have been tested in the clinic. However, their translation from experimental to clinical studies for improving patient outcomes has been both challenging and disappointing [65]. Moreover, preclinical trials often involve young, healthy animals, which do not account for factors like aging or other chronic conditions that significantly impact the outcomes in human patients [26,66].

The results of human studies have been mixed, with some showing no effect (beta-blockers, remote ischemic conditioning [67], Cangrelor [68]), and some showing promising results (colchicine [69], interleukin 1 blockade [70], Canakinumab [71]). In most cases, it was only a partial influence on one of the complex mechanisms of IR damage, not a comprehensive treatment of IR damage.

Sex hormones and sex-specific signaling pathways also play an important role in modulating their efficacy in the presence of comorbidities on animal models, though some findings indicate that comorbidities can differentially affect IRI between sexes; this field remains till now underexplored. Most preclinical studies focus on healthy animals, often neglecting sex and comorbidity influences and very often neglecting the combination of sex and comorbidities.

Incorporating both sexes and relevant comorbidities in experimental models is essential for a comprehensive understanding of biological responses to IR injury [72].

Several problems can arise when applying conditioning. Activation of tolerance can be prevented using antioxidants [73], proteasome inhibitors [74], or other substances such as naloxone [75], and high glucose concentrations also prevent the development of tolerance [76]. On the other hand, the use of antioxidants—by preventing the development of tolerance—could significantly interfere with the treatment of diseases where we aim to eliminate unwanted cells from the body.

Metabolic syndrome (MetS), a cluster of conditions including abdominal obesity, dyslipidemia, hypertension, metainflammation, and glucose intolerance, represents a multi-pronged assault on cardioprotective mechanisms. The effect of conditioning decreases with age and with the presence of diseases, including diabetes [77,78], high cholesterol [79], and obesity [80].

**Figure 3:**
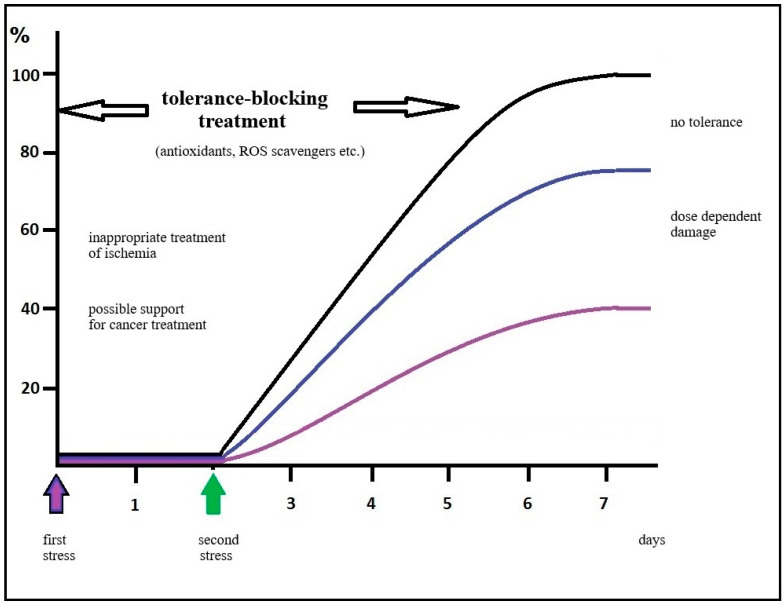
A situational diagram in the case of inappropriate treatment of insults as shown in Figure 1. The use of antioxidants, ROS scavengers, protein synthesis inhibitors, or possibly inhibitors of specific enzyme activity can prevent the development of tolerance. On the other hand, preventing the activation of tolerance can significantly enhance the outcomes of this treatment [73,74,75].

Based on the mentioned results, use of antioxidants, protein synthesis inhibitors, or inhibitors of specific enzyme activity can prevent the development of tolerance; on the other hand, preventing (blocking) the activation of tolerance, which inevitably occurs during cancer treatment, could significantly enhance the outcomes of this treatment (Figure 3).

**Figure 4:**
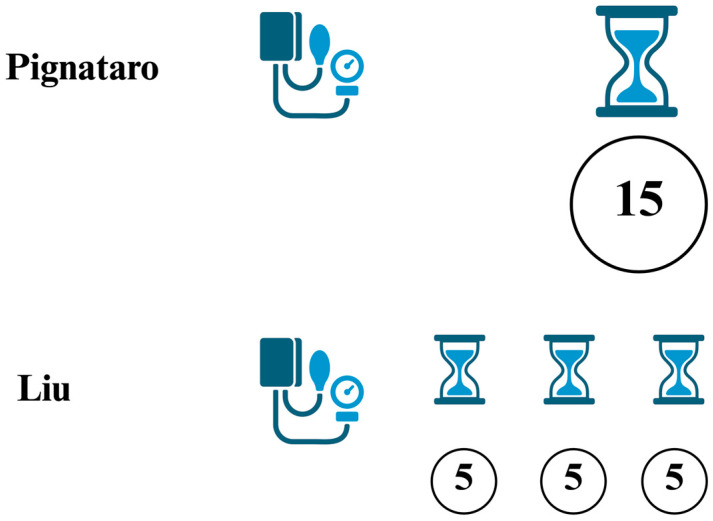
An example of conditioning methods practiced by applying an inflatable cuff to the limb. Created in BioRender. Burda, R. (2025) https://BioRender.com/5j1d6v8.

Remote conditioning shows that there is no need to develop tissue-specific conditioning methods (e.g., neuro-conditioning or cardio-conditioning). A comparison of conditioning methods seems more favorable for remote conditioning, practiced by applying an inflatable cuff to the limb. Here, the question arises whether to use one longer interval of ischemia, as described by Pignataro [81], or to apply more (most often three) cycles of shorter intervals interspersed with roughly equal reperfusion intervals, as described by Liu [82] (Figure 4).

The essential condition for the use of postconditioning is that it must be applied in a way that is both safe and acceptable for the patient (i.e., the patient can tolerate the tourniquet application time). Postconditioning can be used at any time after lethal stress; it can also be applied repeatedly over several days and, of course, can be combined with other treatment procedures. A method that reliably induces tolerance in elderly, ill, and medicated patients would be invaluable.

Another important problem is the reproducibility of the results of individual studies, which is why the IMPACT studies represent a significant challenge. Numerous cardioprotective interventions have been reported to reduce myocardial infarct size (IS) in pre-clinical studies. However, their translation for the benefit of patients with acute myocardial infarction (AMI) has been largely disappointing. One reason for the lack of translation is the lack of rigor and reproducibility in pre-clinical studies. Pig AMI multicenter European network with centralized randomization and core blinded IS analysis was established and validated with the aim to improve the reproducibility of cardioprotective interventions in pre-clinical studies and the translation of cardioprotection for patient benefit. It was performed in a similar way in another study on small animals (rodents and rabbits) [83,84].

## 5. Future Perspectives

Logically, further research continues to identify the key mechanisms and substances responsible for ensuring tolerance. On the other hand, it has been proposed to begin applying the knowledge gained so far, particularly regarding the spread of tolerance effectors via the humoral route (proofed in animal model) [5] and their transfer in the form of blood derivatives from young, healthy, unmedicated donors to generate neuroprotective plasma.

It can be expected that the triggers of ischemic tolerance are contained in plasma; they are probably low-molecular hydrophobic substances and are independent of local neurogenic activity and require activation of local opioid receptors [5].

Since the effectiveness of remote ischemic postconditioning has also been proven in the case of delayed neuronal death induced by temporary global brain ischemia or kainate intoxication (animal model), it can be assumed that the conditioning products are able to overcome the blood–brain barrier [62].

The effect of preconditioned plasma (activated plasma) was already demonstrated by Zhao [85]. Preconditioned plasma (collected from donor animals 48 h after limb ischemia) was able to reduce the extent of IR myocardial damage after transfer to recipients, which was clinically manifested by a reduction in the incidence and duration of ventricular tachycardia. A very similar result was also achieved by Weber [86], who by applying ischemic preconditioned plasma to volunteers achieved protection of human umbilical endothelial cells from hypoxia-induced damage. In the mentioned case, the plasma was taken away directly after finishing preconditioning. Weber and Zhao in all the above cases made only a transfer of “partially” activated plasma. The plasma was activated only by one stress, meaning that only ischemic tolerance triggers were present in it. On the other hand, in our experiments, double-activated plasma (exposure of animals to 2 stresses within a time of 48 h, plasma was collected 6 h after the second stress) was used. By collecting double-activated plasma, effectors, not ischemic tolerance triggers, should be included in plasma. The advantage of double-activated (“fully activated”) plasma containing complete activated ischemic tolerance also lies in the fact that comedication and comorbidity in the recipient will probably have no effect on its immediate effectiveness.

Unless there is a clear chemical identification of the effectors of ischemic tolerance, the simplest and easiest way to activate ischemic tolerance will be to prepare activated plasma from young healthy donors with the possibility of its immediate use in recipients during initial treatment. This does not exclude repeated administration and simultaneous use of other treatment methods.

Based on our experimental work on animal models [87,88,89], we recommend the following applicable timeline for the preparation of activated plasma, based on published work:

Day 1: Initial conditioning (e.g., a single 10–20 min limb ischemia session or three cycles of 5–8 min of cuff inflation/deflation). A subsequent 48 h period of “reperfusion” allows activation of the first stage of tolerance and accumulation of signaling molecules ready for a rapid response to the second stimulus [90].

**Figure 5:**
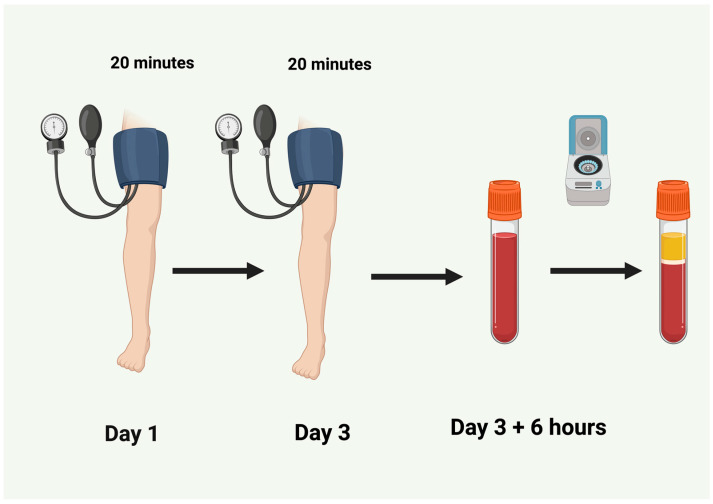
An applicable timeline for the future preparation of double-activated plasma based on published works. Created in BioRender. Burda, R. (2025) https://BioRender.com/nf6tp0t.

Day 3: Second conditioning (using the same protocol as on Day 1). Blood collection follows 6 h, allowing time for synthesis or activation (via posttranslational modifications) of tolerance effectors (Figure 5).

The above-mentioned usage of activated plasma has not been performed in the clinical trials yet and is just a hypothetical scheme suggested by the authors.

Based on our experimental work on animal models, it is expected that blood plasma should be effective immediately after administration into the patient’s bloodstream, but it should also retain its activity after 30 days of storage at −80 degrees Celsius, as well as after lyophilization. It has been shown on animal models to be effective not only in cerebral ischemia but also in 3MT (3-methyltin) intoxication [87]. Due to its conditioning effect, it has also shown potential in protecting against other toxic substances such as kainate in the brain [60,62,64], and doxorubicin [91] and isoprenaline [92] in the heart and muscle.

## 6. Conclusions

Anoxic, ischemic, traumatic, and toxic brain damage represent a huge individual and family tragedy, as well as a big economic burden. The use of current therapy means long-term mild improvement with lifelong consumption of an expensive medication.

The difference between the treatments used in clinics and the potential use of ischemic tolerance is incomparable. Tolerance allows surviving multiple lethal doses, and it is a rapid and inexpensive technique.

While most of the methods used so far aim to mitigate the damage that has already occurred, ischemic tolerance prevents this damage by utilizing the phenomenon of delayed neuronal death.

The concept of “active plasma” holds enormous potential for emergency medicine and the prevention of neurodegeneration, and it may lead to the first universal neuroprotective therapy based on humoral factors.

## Data Availability

No new data were created or analyzed in this study. Data sharing is not applicable to this article.

## References

[1] Murry C.E., Jennings R.B., Reimer K.A. (1986). Preconditioning with ischemia: A delay of lethal cell injury in ischemic myocardium. Circulation.

[2] Gidday J.M. (2006). Cerebral preconditioning and ischaemic tolerance. Nat. Rev. Neurosci..

[3] Zemke D., Smith J.L., Reeves M.J., Majid A. (2004). Ischemia and ischemic tolerance in the brain: An overview. Neurotoxicology.

[4] Przyklenk K., Bauer B., Ovize M., Kloner R.A., Whittaker P. (1993). Regional ischemic ‘preconditioning’ protects remote virgin myocardium from subsequent sustained coronary occlusion. Circulation.

[5] Shimizu M., Tropak M., Diaz R.J., Suto F., Surendra H., Kuzmin E., Li J., Gross G., Wilson G.J., Callahan J. (2009). Transient limb ischaemia remotely preconditions through a humoral mechanism acting directly on the myocardium: Evidence suggesting cross-species protection. Clin. Sci..

[6] Siesjo B.K., Katsura K.I., Kristian T., Li P.A., Siesjo P. (1996). Molecular mechanisms of acidosis-mediated damage. Acta Neurochir. Suppl..

[7] Danielisova V., Gottlieb M., Nemethova M., Kravcukova P., Domorakova I., Mechirova E., Burda J. (2009). Bradykinin postconditioning protects pyramidal CA1 neurons against delayed neuronal death in rat hippocampus. Cell. Mol. Neurobiol..

[8] Kirino T. (1982). Delayed neuronal death in the gerbil hippocampus following ischemia. Brain Res..

[9] Kirino T., Tamura A., Sano K. (1984). Delayed neuronal death in the rat hippocampus following transient forebrain ischemia. Acta Neuropathol..

[10] Vanlangenakker N., Vanden Berghe T., Krysko D.V., Festjens N., Vandenabeele P. (2008). Molecular mechanisms and pathophysiology of necrotic cell death. Curr. Mol. Med..

[11] Dumont E.A., Reutelingsperger C.P., Smits J.F., Daemen M.J., Doevendans P.A., Wellens H.J., Hofstra L. (2001). Real-time imaging of apoptotic cell-membrane changes at the single-cell level in the beating murine heart. Nat. Med..

[12] Gottlieb R.A., Burleson K.O., Kloner R.A., Babior B.M., Engler R.L. (1994). Reperfusion injury induces apoptosis in rabbit cardiomyocytes. J. Clin. Investig..

[13] Anversa P., Cheng W., Liu Y., Leri A., Redaelli G., Kajstura J. (1998). Apoptosis and myocardial infarction. Basic Res. Cardiol..

[14] Zhao Z.Q., Nakamura M., Wang N.P., Wilcox J.N., Shearer S., Ronson R.S., Guyton R.A., Vinten-Johansen J. (2000). Reperfusion induces myocardial apoptotic cell death. Cardiovasc. Res..

[15] Berges A., Van Nassauw L., Bosmans J., Timmermans J.P., Vrints C. (2003). Role of nitric oxide and oxidative stress in ischaemic myocardial injury and preconditioning. Acta Cardiol..

[16] Granger D.N., Kvietys P.R. (2015). Reperfusion injury and reactive oxygen species: The evolution of a concept. Redox Biol..

[17] Xiao J., Liang D., Zhang H., Liu Y., Li F., Chen Y.H. (2010). 4′-Chlorodiazepam, a translocator protein (18 kDa) antagonist, improves cardiac functional recovery during postischemia reperfusion in rats. Exp. Biol. Med..

[18] Zweier J.L., Talukder M.A. (2006). The role of oxidants and free radicals in reperfusion injury. Cardiovasc. Res..

[19] Steenbergen C., Fralix T.A., Murphy E. (1993). Role of increased cytosolic free calcium concentration in myocardial ischemic injury. Basic Res. Cardiol..

[20] Hausenloy D.J., Ong S.B., Yellon D.M. (2009). The mitochondrial permeability transition pore as a target for preconditioning and postconditioning. Basic Res. Cardiol..

[21] Bodsch W., Barbier A., Oehmichen M., Grosse Ophoff B., Hossmann K.A. (1986). Recovery of monkey brain after prolonged ischemia. II. Protein synthesis and morphological alterations. J. Cereb. Blood Flow Metab..

[22] Burda J., Martin M.E., Garcia A., Alcazar A., Fando J.L., Salinas M. (1994). Phosphorylation of the alpha subunit of initiation factor 2 correlates with the inhibition of translation following transient cerebral ischaemia in the rat. Biochem. J..

[23] Nemethova M., Danielisova V., Gottlieb M., Kravcukova P., Burda J. (2010). Ischemic postconditioning in the rat hippocampus: Mapping of proteins involved in reversal of delayed neuronal death. Arch. Ital. Biol..

[24] Zhang C., Tang Y., Li Y., Xie L., Zhuang W., Liu J., Gong J. (2017). Unfolded protein response plays a critical role in heart damage after myocardial ischemia/reperfusion in rats. PLoS ONE.

[25] Wang L., Liu Y., Zhang X., Ye Y., Xiong X., Zhang S., Gu L., Jian Z., Wang H. (2022). Endoplasmic Reticulum Stress and the Unfolded Protein Response in Cerebral Ischemia/Reperfusion Injury. Front. Cell. Neurosci..

[26] Ghanta S.N., Kattamuri L.P.V., Odueke A., Mehta J.L. (2025). Molecular Insights into Ischemia-Reperfusion Injury in Coronary Artery Disease: Mechanisms and Therapeutic Implications: A Comprehensive Review. Antioxidants.

[27] Gulyaeva N.V. (2021). Does the inability of CA1 area to respond to ischemia with early rapid adenosine release contribute to hippocampal vulnerability?: An Editorial Highlight for “Spontaneous, transient adenosine release is not enhanced in the CA1 region of hippocampus during severe ischemia models”. J. Neurochem..

[28] Popov S.V., Mukhomedzyanov A.V., Voronkov N.S., Derkachev I.A., Boshchenko A.A., Fu F., Sufianova G.Z., Khlestkina M.S., Maslov L.N. (2023). Regulation of autophagy of the heart in ischemia and reperfusion. Apoptosis.

[29] Chen S., Li S. (2012). The Na^+^/Ca^2+^ exchanger in cardiac ischemia/reperfusion injury. Med. Sci. Monit..

[30] Kolnik S., Corry K., Hildahl K., Filteau J., White O., Brandon O., Farid L., Shearlock A., Moralejo D., Juul S.E. (2022). Vitamin E Decreases Cytotoxicity and Mitigates Inflammatory and Oxidative Stress Responses in a Ferret Organotypic Brain Slice Model of Neonatal Hypoxia-Ischemia. Dev. Neurosci..

[31] Masereel B., Pochet L., Laeckmann D. (2003). An overview of inhibitors of Na(+)/H(+) exchanger. Eur. J. Med. Chem..

[32] Gottlieb M., Wang Y., Teichberg V.I. (2003). Blood-mediated scavenging of cerebrospinal fluid glutamate. J. Neurochem..

[33] Nagy D., Marosi M., Kis Z., Farkas T., Rakos G., Vecsei L., Teichberg V.I., Toldi J. (2009). Oxaloacetate decreases the infarct size and attenuates the reduction in evoked responses after photothrombotic focal ischemia in the rat cortex. Cell. Mol. Neurobiol..

[34] Beyersdorf F., Schlensak C. (2009). Controlled reperfusion after acute and persistent limb ischemia. Semin. Vasc. Surg..

[35] Zeineddine H.A., Hong S.H., Peesh P., Dienel A., Torres K., Thankamani Pandit P., Matsumura K., Huang S., Li W., Chauhan A. (2024). Neutrophils and Neutrophil Extracellular Traps Cause Vascular Occlusion and Delayed Cerebral Ischemia After Subarachnoid Hemorrhage in Mice. Arterioscler. Thromb. Vasc. Biol..

[36] Bull D.A., Maurer J. (2003). Aprotinin and preservation of myocardial function after ischemia-reperfusion injury. Ann. Thorac. Surg..

[37] Peralta-Leal A., Rodriguez-Vargas J.M., Aguilar-Quesada R., Rodriguez M.I., Linares J.L., de Almodovar M.R., Oliver F.J. (2009). PARP inhibitors: New partners in the therapy of cancer and inflammatory diseases. Free Radic. Biol. Med..

[38] Li J., Zhang Y., Tang R., Liu H., Li X., Lei W., Chen J., Jin Z., Tang J., Wang Z. (2024). Glycogen synthase kinase-3beta: A multifaceted player in ischemia-reperfusion injury and its therapeutic prospects. J. Cell. Physiol..

[39] Tang D., Xu C., Jiang Z., Meng Z., Zhang M., Fan F., Liu H. (2025). OTSSP167 suppresses TNBC brain metastasis via ROS-driven P38/JNK and FAK/ERK pathways. Eur. J. Pharmacol..

[40] Zajaczkowski S., Ziolkowski W., Badtke P., Zajaczkowski M.A., Flis D.J., Figarski A., Smolinska-Bylanska M., Wierzba T.H. (2018). Promising effects of xanthine oxidase inhibition by allopurinol on autonomic heart regulation estimated by heart rate variability (HRV) analysis in rats exposed to hypoxia and hyperoxia. PLoS ONE.

[41] Sylvester A.L., Zhang D.X., Ran S., Zinkevich N.S. (2022). Inhibiting NADPH Oxidases to Target Vascular and Other Pathologies: An Update on Recent Experimental and Clinical Studies. Biomolecules.

[42] Fragasso G., Salerno A., Lattuada G., Cuko A., Calori G., Scollo A., Ragogna F., Arioli F., Bassanelli G., Spoladore R. (2011). Effect of partial inhibition of fatty acid oxidation by trimetazidine on whole body energy metabolism in patients with chronic heart failure. Heart.

[43] Padron-Barthe L., Villalba-Orero M., Gomez-Salinero J.M., Dominguez F., Roman M., Larrasa-Alonso J., Ortiz-Sanchez P., Martinez F., Lopez-Olaneta M., Bonzon-Kulichenko E. (2019). Severe Cardiac Dysfunction and Death Caused by Arrhythmogenic Right Ventricular Cardiomyopathy Type 5 Are Improved by Inhibition of Glycogen Synthase Kinase-3beta. Circulation.

[44] Zhao Z., Chen X., Zhuang Z., Chen J., Zheng Z., Wu W., Wu Z., Lin Q., Chen M., Liu J. (2025). Exercise Training Promotes Neural Remodeling and Vascular Regeneration in Cerebral Ischemic Rats Through the JNK/c-jun Signaling Pathway. Mol. Neurobiol..

[45] Akoumianakis I., Polkinghorne M., Antoniades C. (2022). Non-canonical WNT signalling in cardiovascular disease: Mechanisms and therapeutic implications. Nat. Rev. Cardiol..

[46] Dong P., Liu K., Han H. (2022). The Role of NF-kappaB in Myocardial Ischemia/Reperfusion Injury. Curr. Protein Pept. Sci..

[47] Egana-Gorrono L., Lopez-Diez R., Yepuri G., Ramirez L.S., Reverdatto S., Gugger P.F., Shekhtman A., Ramasamy R., Schmidt A.M. (2020). Receptor for Advanced Glycation End Products (RAGE) and Mechanisms and Therapeutic Opportunities in Diabetes and Cardiovascular Disease: Insights from Human Subjects and Animal Models. Front. Cardiovasc. Med..

[48] Cui X., He Z., Liang Z., Chen Z., Wang H., Zhang J. (2017). Exosomes from Adipose-derived Mesenchymal Stem Cells Protect the Myocardium Against Ischemia/Reperfusion Injury Through Wnt/beta-Catenin Signaling Pathway. J. Cardiovasc. Pharmacol..

[49] Norouzi-Barough L., Shirian S., Gorji A., Sadeghi M. (2022). Therapeutic potential of mesenchymal stem cell-derived exosomes as a cell-free therapy approach for the treatment of skin, bone, and cartilage defects. Connect. Tissue Res..

[50] Li Z., He F., Dai S., Yu Q., Si C., Wu F., Zhao W., Zhang B., Xu P. (2025). Exosomes derived from human umbilical cord mesenchymal stem cells ameliorate AKI after cardiac surgery by facilitating miR-21-5p targeting TEAD1. Ren. Fail..

[51] Theofilis P., Oikonomou E., Vogiatzi G., Antonopoulos A.S., Siasos G., Iliopoulos D.C., Perrea D., Tsioufis C., Tousoulis D. (2021). The impact of proangiogenic microRNA modulation on blood flow recovery following hind limb ischemia. A systematic review and meta-analysis of animal studies. Vasc. Pharmacol..

[52] Li H., Wei C., Gao J., Bai S., Li H., Zhao Y., Li H., Han L., Tian Y., Yang G. (2014). Mediation of dopamine D2 receptors activation in post-conditioning-attenuated cardiomyocyte apoptosis. Exp. Cell Res..

[53] Gomez L., Paillard M., Thibault H., Derumeaux G., Ovize M. (2008). Inhibition of GSK3beta by postconditioning is required to prevent opening of the mitochondrial permeability transition pore during reperfusion. Circulation.

[54] Chen H., Shen J., Zhao H. (2020). Ischemic postconditioning for stroke treatment: Current experimental advances and future directions. Cond. Med..

[55] Penna C., Granata R., Tocchetti C.G., Gallo M.P., Alloatti G., Pagliaro P. (2015). Endogenous Cardioprotective Agents: Role in Pre and Postconditioning. Curr. Drug Targets.

[56] Burda J., Danielisova V., Nemethova M., Gottlieb M., Matiasova M., Domorakova I., Mechirova E., Ferikova M., Salinas M., Burda R. (2006). Delayed postconditionig initiates additive mechanism necessary for survival of selectively vulnerable neurons after transient ischemia in rat brain. Cell. Mol. Neurobiol..

[57] Pignataro G., Brancaccio P., Laudati G., Valsecchi V., Anzilotti S., Casamassa A., Cuomo O., Vinciguerra A. (2020). Sodium/calcium exchanger as main effector of endogenous neuroprotection elicited by ischemic tolerance. Cell Calcium.

[58] Pignataro G. (2021). Emerging Role of microRNAs in Stroke Protection Elicited by Remote Postconditioning. Front. Neurol..

[59] Burda J., Matiasova M., Gottlieb M., Danielisova V., Nemethova M., Garcia L., Salinas M., Burda R. (2005). Evidence for a role of second pathophysiological stress in prevention of delayed neuronal death in the hippocampal CA1 region. Neurochem. Res..

[60] Burda J., Danielisova V., Nemethova M., Gottlieb M., Kravcukova P., Domorakova I., Mechirova E., Burda R. (2009). Postconditioning and anticonditioning: Possibilities to interfere to evoked apoptosis. Cell. Mol. Neurobiol..

[61] Danielisova V., Gottlieb M., Nemethova M., Burda J. (2008). Effects of bradykinin postconditioning on endogenous antioxidant enzyme activity after transient forebrain ischemia in rat. Neurochem. Res..

[62] Burda R., Danielisova V., Gottlieb M., Nemethova M., Bonova P., Matiasova M., Morochovic R., Burda J. (2014). Delayed remote ischemic postconditioning protects against transient cerebral ischemia/reperfusion as well as kainate-induced injury in rats. Acta Histochem..

[63] Herpich M.E., de Oliveira Guarnieri L., de Oliveira A.C.P., Moraes M.F.D. (2024). Bacterial Lipopolysaccharide Post-Conditioning in The kainic acid animal model of Temporal Lobe epilepsy. Epilepsy Behav..

[64] Nagy D., Kocsis K., Fuzik J., Marosi M., Kis Z., Teichberg V.I., Toldi J., Farkas T. (2011). Kainate postconditioning restores LTP in ischemic hippocampal CA1: Onset-dependent second pathophysiological stress. Neuropharmacology.

[65] Hausenloy D.J., Garcia-Dorado D., Botker H.E., Davidson S.M., Downey J., Engel F.B., Jennings R., Lecour S., Leor J., Madonna R. (2017). Novel targets and future strategies for acute cardioprotection: Position Paper of the European Society of Cardiology Working Group on Cellular Biology of the Heart. Cardiovasc. Res..

[66] Roolvink V., Ibanez B., Ottervanger J.P., Pizarro G., van Royen N., Mateos A., Dambrink J.E., Escalera N., Lipsic E., Albarran A. (2016). Early Intravenous Beta-Blockers in Patients with ST-Segment Elevation Myocardial Infarction Before Primary Percutaneous Coronary Intervention. J. Am. Coll. Cardiol..

[67] Garcia Del Blanco B., Otaegui I., Rodriguez-Palomares J.F., Bayes-Genis A., Fernandez-Nofrerias E., Vilalta Del Olmo V., Carrillo X., Ibanez B., Worner F., Casanova J. (2021). Effect of COMBinAtion therapy with remote ischemic conditioning and exenatide on the Myocardial Infarct size: A two-by-two factorial randomized trial (COMBAT-MI). Basic Res. Cardiol..

[68] Bulluck H., Chong J.H., Bryant J., Annathurai A., Chai P., Chan M., Chawla A., Chin C.Y., Chung Y.C., Gao F. (2024). Effect of Cangrelor on Infarct Size in ST-Segment-Elevation Myocardial Infarction Treated by Primary Percutaneous Coronary Intervention: A Randomized Controlled Trial (The PITRI Trial). Circulation.

[69] Nidorf S.M., Fiolet A.T.L., Mosterd A., Eikelboom J.W., Schut A., Opstal T.S.J., The S.H.K., Xu X.F., Ireland M.A., Lenderink T. (2020). Colchicine in Patients with Chronic Coronary Disease. N. Engl. J. Med..

[70] Abbate A., Wohlford G.F., Del Buono M.G., Chiabrando J.G., Markley R., Turlington J., Kadariya D., Trankle C.R., Biondi-Zoccai G., Lipinski M.J. (2022). Interleukin-1 blockade with anakinra and heart failure following ST-segment elevation myocardial infarction: Results from a pooled analysis of the VCUART clinical trials. Eur. Heart J. Cardiovasc. Pharmacother..

[71] Ridker P.M., Everett B.M., Thuren T., MacFadyen J.G., Chang W.H., Ballantyne C., Fonseca F., Nicolau J., Koenig W., Anker S.D. (2017). Antiinflammatory Therapy with Canakinumab for Atherosclerotic Disease. N. Engl. J. Med..

[72] Rocca C., Femmino S., Aquila G., Granieri M.C., De Francesco E.M., Pasqua T., Rigiracciolo D.C., Fortini F., Cerra M.C., Maggiolini M. (2018). Notch1 Mediates Preconditioning Protection Induced by GPER in Normotensive and Hypertensive Female Rat Hearts. Front. Physiol..

[73] Puisieux F., Deplanque D., Bulckaen H., Maboudou P., Gele P., Lhermitte M., Lebuffe G., Bordet R. (2004). Brain ischemic preconditioning is abolished by antioxidant drugs but does not up-regulate superoxide dismutase and glutathion peroxidase. Brain Res..

[74] Dosenko V.E., Nagibin V.S., Tumanovskaya L.V., Zagoriy V.Y., Moibenko A.A., Vaage J. (2006). Proteasome inhibitors eliminate protective effect of postconditioning in cultured neonatal cardiomyocytes. Fiziol. Zhurnal.

[75] Dickson E.W., Blehar D.J., Carraway R.E., Heard S.O., Steinberg G., Przyklenk K. (2001). Naloxone blocks transferred preconditioning in isolated rabbit hearts. J. Mol. Cell. Cardiol..

[76] Yu J., Maimaitili Y., Xie P., Wu J.J., Wang J., Yang Y.N., Ma H.P., Zheng H. (2017). High glucose concentration abrogates sevoflurane post-conditioning cardioprotection by advancing mitochondrial fission but dynamin-related protein 1 inhibitor restores these effects. Acta Physiol..

[77] Gao S., Yang Z., Shi R., Xu D., Li H., Xia Z., Wu Q.P., Yao S., Wang T., Yuan S. (2016). Diabetes blocks the cardioprotective effects of sevoflurane postconditioning by impairing Nrf2/Brg1/HO-1 signaling. Eur. J. Pharmacol..

[78] Tyagi S., Singh N., Virdi J.K., Jaggi A.S. (2019). Diabetes abolish cardioprotective effects of remote ischemic conditioning: Evidences and possible mechanisms. J. Physiol. Biochem..

[79] Wen C., Xue F.S., Wang Y.H., Jin J.H., Liao X. (2022). Hypercholesterolemia attenuates cardioprotection of ischemic preconditioning and postconditioning with alpha7 nicotinic acetylcholine receptor agonist by enhancing inflammation and inhibiting the PI3K/Akt/eNOS pathway. Exp. Ther. Med..

[80] Yang Z., Chen Y., Zhang Y., Jiang Y., Fang X., Xu J. (2014). Sevoflurane postconditioning against cerebral ischemic neuronal injury is abolished in diet-induced obesity: Role of brain mitochondrial KATP channels. Mol. Med. Rep..

[81] Pignataro G., Esposito E., Sirabella R., Vinciguerra A., Cuomo O., Di Renzo G., Annunziato L. (2013). nNOS and p-ERK involvement in the neuroprotection exerted by remote postconditioning in rats subjected to transient middle cerebral artery occlusion. Neurobiol. Dis..

[82] Liu K., Cai Z., Zhang Q., He J., Cheng Y., Wei S., Yin M. (2022). Determination of significant parameters in remote ischemic postconditioning for ischemic stroke in experimental models: A systematic review and meta-analysis study. CNS Neurosci. Ther..

[83] Kleinbongard P., Arriola C.G., Badimon L., Crisostomo V., Giricz Z., Gyongyosi M., Heusch G., Ibanez B., Kiss A., de Kleijn D.P.V. (2024). The IMproving Preclinical Assessment of Cardioprotective Therapies (IMPACT): Multicenter pig study on the effect of ischemic preconditioning. Basic Res. Cardiol..

[84] Hernandez-Resendiz S., Vilskersts R., Aluja D., Andreadou I., Bencsik P., Dambrova M., Efentakis P., Gao F., Giricz Z., Brenner G.B. (2025). Correction: IMproving Preclinical Assessment of Cardioprotective Therapies (IMPACT): A small animal acute myocardial infarction randomized-controlled multicenter study on the effect of ischemic preconditioning. Basic Res. Cardiol..

[85] Zhao Y., Zheng Z.N., Liu X., Dai G., Jin S.Q. (2018). Effects of preconditioned plasma collected during the late phase of remote ischaemic preconditioning on ventricular arrhythmias caused by myocardial ischaemia reperfusion in rats. J. Int. Med. Res..

[86] Weber N.C., Riedemann I., Smit K.F., Zitta K., van de Vondervoort D., Zuurbier C.J., Hollmann M.W., Preckel B., Albrecht M. (2015). Plasma from human volunteers subjected to remote ischemic preconditioning protects human endothelial cells from hypoxia-induced cell damage. Basic Res. Cardiol..

[87] Burda R., Danielisová V., Burda J. (2019). The End Effector of Ischemic Tolerance Present in Blood Plasma from Double Conditioned Donors Ameliorates Trimethyltin Provoked Damage in Brain. OBM Neurobiol..

[88] Burda R., Morochovic R., Nemethova M., Burda J. (2020). Remote ischemic postconditioning as well as blood plasma from double-conditioned donor ameliorate reperfusion syndrome in skeletal muscle. J. Plast. Surg. Hand Surg..

[89] Burda R., Burda J., Morochovic R. (2023). Ischemic Tolerance-A Way to Reduce the Extent of Ischemia-Reperfusion Damage. Cells.

[90] Gidday J.M. (2015). Extending injury- and disease-resistant CNS phenotypes by repetitive epigenetic conditioning. Front. Neurol..

[91] Lee Y., Kwon I., Jang Y., Cosio-Lima L., Barrington P. (2020). Endurance Exercise Attenuates Doxorubicin-induced Cardiotoxicity. Med. Sci. Sports Exerc..

[92] Wong S.M., Chiu P.Y., Leung H.Y., Zhou L., Zuo Z., Lam P.Y., Ko K.M. (2011). Myocardial post-conditioning with Danshen-Gegen decoction protects against isoproterenol-induced myocardial injury via a PKCepsilon/mKATP-mediated pathway in rats. Chin. Med..

